# Effects of different tooth grinding procedures on the occurrence of tooth injuries, skin lesions, performance and behaviour of suckling piglets

**DOI:** 10.1186/s40813-024-00373-x

**Published:** 2024-06-12

**Authors:** Franziska Anna kleine Kruthaup, Michaela Fels, Carolin Bernarda Timphaus, Fritjof Freise, Swetlana Herbrandt, Elisabeth große Beilage

**Affiliations:** 1 Veterinary Practice Dümmerland, Bahnhofstraße 40, 49439 Steinfeld, Field, Germany; 2 Field Station for Epidemiology in Bakum, University of Veterinary Medicine Hannover, Büscheler Straße 9, 49456 Bakum, Germany; 3 Institute of Animal Hygiene, Animal Welfare and Farm Animal Behaviour, University of Veterinary Medicine Hannover, Bischofshofer Damm 15, 30173 Foundation, Hannover, Germany; 4 Veterinary Practice Am Brettberg, Steinfelder Straße 28.a, 49393 Lohne, Germany; 5 Department of Biometry, Epidemiology and Information Processing, University of Veterinary Medicine Hannover, Foundation, Bünteweg 2, 30559 Hannover, Germany; 6 Statistical Consulting and Analysis, TU Dortmund University, Vogelpothsweg 78, 44227 Dortmund, Germany

**Keywords:** Tea-cup, Sow, Udder, Facial lesion, Sow teat lesion

## Abstract

**Background:**

Immediately after birth, newborn piglets fight to establish a teat order. During this process, lesions appear on the piglets’ faces and on the sows’ teats, which is why tooth resection is carried out on many farms in Germany even though it is known that this procedure is frequently resulting in pulp openings. The opening of a pulp cave is suspected to cause painful tooth alterations and may be an entrance for infectious agents. The purpose of this study was to analyse the effect of tooth resection on skin lesions, development of bodyweight and behaviour in suckling piglets. Four days prepartum, 110 sows in farrow-to-finish production were assigned to one of three treatments. Litters had their teeth left intact (control group, CG), ground with a tea-cup roller head (Tea-cup head grinder group, TCG, Wilofa Diamant, D-56,133 Fachbach, Germany) or ground with a diamond rolling head (rolling head grinder group, RG, IBS/E Company Proxxon GmbH, 54,343 Föhren, Germany). The number of pulp openings in the RG and TCG was examined using a random sample. Piglet body weight and skin lesion scores were recorded within the first 24 h after birth and during each week of the suckling period. Each sow’s udder was examined before farrowing, in the second week of lactation and at weaning. The behaviour of the litters from nine sows was video-recorded throughout the suckling period. The aim of this study was to investigate the effects of tooth grinding by a tea-cup head (compared to grinding by a diamond roller head and no grinding [control group]) on the behaviour and average daily gain of piglets as well as on skin lesions on sow udder.

**Results:**

The number of dental injuries was significantly greater in the RG than in the TCG (*p* < 0.01). Head lesions on piglets were significantly more common in the CG than in the RG (*p* = 0. 02). Compared to CG piglets, TCG piglets had a significantly greater weight at the end of the suckling period (*p* = 0.02). No significant difference between treatments was found in the sows’ udder (parenchyma, skin, or teat) or in the behaviour of the litters.

**Conclusion:**

As tooth grinding is frequently inducing pulp openings, the necessity of the procedure should be carefully and critically scrutinised. In case tooth resection seems inevitable until the underlying management problems have been solved, the Tea-cup grinding head should be used due to significantly fewer pulp openings.

## Background

Piglets are born with a total of 28 intact deciduous teeth. In particular, the tips of decidual incisor 3 (Id3) and the decidual canine (Cd) have sharp edges that may hurt the litter mates or the sow’s udder immediately after birth during the fight for access to teats [1, 2]. Tooth resection, i.e., the shortening of the tips of Id3 and Cd, is a procedure routinely performed by many farmers on piglets within the first days of life [3]. The procedure is aimed at reducing facial lesions in piglets during the establishment of the ´teat order` [4, 5] as well as reducing lesions on the sow’s udder [6, 7]. However, the central issue of common tooth grinding methods is that the procedure is suspected being painful [8] and frequently resulting in the opening of the pulp cavity [9]. The opening of a pulp cavity increases the risk of subsequent infection [10] and is an indication for immediate dental filling in human medicine [11].

In the past, clipping with the aid of a side cutter or special piglet tooth nippers was favoured for tooth resection. With this method, it was impossible to restrict the resection to the tips, and damage to a significant part of the tooth, resulting in fractures, pulpitis and abscesses, was inevitable [12–14]. Currently, grinders with diamond rolling heads are frequently used to remove the tips of teeth. Tooth fractures can be prevented by this method, but the frequent opening of the pulp cavity is still a remaining problem [9].

For animal welfare reasons, in Germany, tooth resection within the first 8 days of life in newborn piglets is only allowed if treatment is necessary to protect the mother or littermates (TierSchG § 5 [1] 5) and according to veterinary indications (TierSchG § 6 [1] 3). Moreover, for the grinding of Id3 and in suckling piglets less than eight days old, there is also an explicit exception for the anaesthesia requirement (TierSchG § 5 [3] 5). Nevertheless, tooth grinding is a common procedure routinely performed in newborn piglets. When tooth grinding is performed, the method must ensure that the tooth surface is smooth and intact after grinding (EU Directive 2008/120/EC Annex I, Chap. 1 [8]). However, this intactness of the tooth surface is not ensured when using a diamond rolling head due to the frequent opening of the pulp cavity [9].

The desired effect associated with shortening the teeth has already been studied in many ways. The different forms of tooth resection (clipping or grinding with a roller head) and their effects on sows and piglets have been investigated. Most studies have shown fewer and/or less-severe skin lesions in tooth-resected piglets [5, 7, 14–16], while Delbor et al. (2000) reported a benefit for clipped piglets at 7 days of age but a disadvantage at 14 and 28 days of age and Hansson et al. (2012) reported no effect of grinding on litter facial lesion scores. Concerning udders, some authors have observed less damage [7, 14] after tooth resection, whereas others have shown no difference between intact and teeth-clipped litters [16, 17]. The studies by Brown et al. (1996) and Delbor et al. (2000) were carried out in outdoor systems, unlike the other investigations. Lesions on the udder were less common in the control group, where the teeth of the piglets were intact. Conclusions concerning the effects of tooth resection on the growth rate of whole litters are also divergent. Researchers have observed either a greater growth rate [14], no difference [5–7, 12, 18] or even a lower growth rate [17, 19] in litters whose teeth were resected. Other studies have shown better weight gain by selective tooth grinding leaving the teeth of smaller piglets intact [20]. Another report suggested that there is greater weight gain in piglets with intact teeth than in piglets with clipped teeth during the period from the third day of life to the end of the first week of life [5]. In addition, one study has reported that there is increased mortality in piglets with intact teeth [14]. This is in contrast to another study in which increased mortality was recorded in piglets with clipped teeth [18]. Six risk factors associated to lesions on the piglets’ faces and the sow’s teats were identified by evaluating various studies. These included the omission of tooth resection, the husbandry system, litter size, piglet management, environmental influences and the sow’s milk production. In addition to the literature review, piglet producers worldwide were asked online to what extent they try to treat or prevent facial lesions while avoiding tooth resection. It was found that on most farms where tooth resection was performed, other potentially effective measures, such as improving the sow’s milk yield or reducing litter size, were not used or were not considered sufficiently effective. In very few cases, tooth resection was only used as a last resort to prevent lesions and therefore alternative measures to control lesions were not tried before resection was introduced, as required by EU legislation [21].

To ensure the legal requirements of a smooth and intact surface of resected teeth, the previously frequently used method of clipping is no longer permitted [14]. Moreover, grinding by the use of diamond rolling heads has to be critically assessed because the surface often cannot be kept intact [9]. This opening of the pulp cavity is caused by inevitably grinding both teeth (Id3 and Cd3) of each quadrant at the same time. The teeth in the upper jaw are of different lengths, with Id3 being longer than Cd3. This inevitably results in opening of the dental pulp of Id3, while only the surface is smoothed in Cd3 [9]. The average thickness of the coronal layer of the dentin and enamel is only 1.3 mm. To ensure that this layer is not opened, tooth resection must basically be performed with great care [9] and each tooth must be grinded individually.

To reduce the risk of opening the pulp cavity by tooth grinding, a so-called Tea-cup grinding head was developed and tested in a field study [22]. Different from the diamond roller head, the Tea-cup head is designed for the grinding of single teeth. The use of the Tea-cup grinding head resulted in a significant reduction in the number of opened pulp cavities by a factor of four compared to that of the conventional diamond roller head [22].

The hypothesis was to find more frequent and more severe skin lesions in the piglets and sows of the control group. Bodyweight was expected to be reduced in piglets with severe skin lesions and active behaviour was also expected to be reduced. Moreover, it was expected that the tooth grinding without cooling is painful and therefore resulting in a short-term modification of behaviour. For the group that underwent tooth grinding with a roller-head, a reduction in bodyweight was expected due to the large number of pulp openings typical for this procedure. Moreover, for these piglets also a (long-term) modification of the behaviour was assumed. In general, the Tea-cup grinder was expected to be superior compared to the roller-head grind and the control group.

## Methods

### Study design

The study was carried out on a commercial pig farm in northwestern Germany, where 1250 sows were kept and managed in a three-week batch farrowing rhythm with a suckling period of 28 days. Each batch comprised 140–170 sows with an average of 2000 piglets (DanBread x PIC 408) born per batch (Table 1). During the period from June to September 2021, the piglets born from 110 sows with an average parity of 2 litters (CG = 2.02, TCG = 2.1, RG = 2.05) in four consecutive farrowing batches were included in the study. Sows were moved to the farrowing pen 6 days before the expected farrowing date and were randomly assigned to the respective treatment groups, where all piglets within the litter underwent the same treatment: CG – control group with intact teeth, TCG – Tea-cup head grinder group, RG – rolling head grinder group. The average litter size was 17,06 life born (CG=17,3; TCG=17,1; RG=16,8) and 1.02 dead born piglets (CG = 1.02; TCG = 1.01; RG = 1.04) per litter. Within 24 h after birth, also litter balancing was performed so that an average of 16 piglets were on the sows at the time of inclusion in the study (CG = 16.35; TCG = 16.25; RG = 15.5). All piglets in a litter were taken into account except those with a birth weight of less than 750 g.

**Table 1:** Examination schedule

Batch	Time period	Total number of sows (*n*)	1st parity (*n*)	≥ 2nd parity (*n*)	Sows per treatment group (*n*)		
					CG*	TCG*	RG*
1	20.06.2021–18.07.2021	33	15	18	11	11	11
2	12.07.2021–08.08.2021	30	9	21	8	12	10
3	31.07.2021–29.08.2021	24	9	15	7	8	9
4	21.08.2021–19.09.2021	23	8	15	8	9	6
Total		110	41	69	34	40	36

* CG = control group; TCG = Tea-cup head grinder group; RG = rolling head grinder group

All piglets were included in the study at birth and followed until weaning (Table 2). The tooth grinding was always performed by the same trained staff and conducted within 24 h after birth (time point 1), the same time, the piglets in all treatment groups received an iron injection, the tail was docked, and each piglet received an ear tag with an individual animal number and a colour indicating the treatment group. The piglets in the CG were not given any sham treatment in addition to the measures mentioned. The day of birth varied for the piglets within one batch for 1to 5 days. For the consecutive data collection (skin scoring, weighing) all piglets born within the same batch were examined the same time on the next Monday (first week of life, time point 2) and again at one week intervals (second/third week of life, time point 3/4). The last examination was carried out on a Sunday, the day before weaning (fourth week of life, time point 5). Mortality during the observation period was evaluated on the basis of the data collected.

**Table 2:** Timeline of the study

Time point	Age of piglets	Tooth resection	Weighing	Skin scoring
1	Birth	X	X	X
2	1 week of life		X	X
3	2 weeks of life		X	X
4	3 weeks of life		X	X
5	4 weeks of life (weaning)		X	X

### Examination of the teeth for damage due to abrasion

**Fig. 1:**
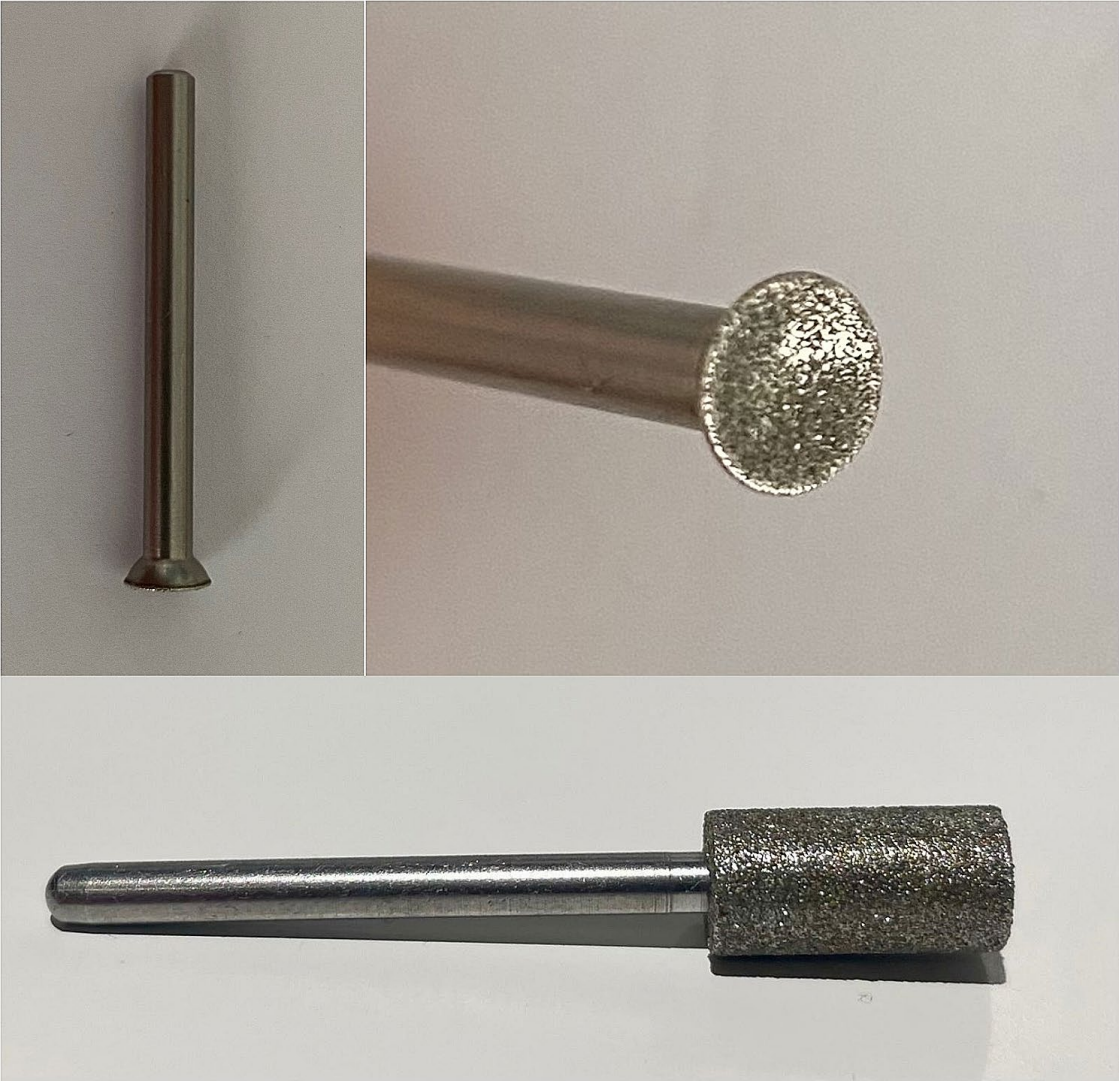
Tea-cup Roller head (above), Roller grinding head (below)

To determine the status quo with regard to the number of opened pulp cavities, the teeth of 79 piglets in each of the treatment groups (RG = rolling head grinder group and TCG = Tea-cup head grinder group) were examined after tooth resection. The teeth (Cd and Id3) of the piglets in the RG group were ground by using an industrial drill grinder (IBS/E, Proxxon GmbH, 54,343 Föhren, Germany). This roller grinding head had a diameter of 7 mm and moved in rotation. A special grinding head was used for grinding the teeth of piglets from the TCG group, and this grinding head was developed by the company Wilofa Diamant, Fachbach, Germany, according to an invention of Hessling - Zeinen (Greven) [22] (Fig. 1). This grinding head has a special concave grinding surface that must be held perpendicular to each tooth individually when grinding.

Pulp openings can be examined by using a dental probe (Eickhoff 2005). Thus, in a separate step, Cd and Id3 of the upper and lower jaw from the piglets were examined individually for pulp opening. Care was taken to fix the piglets gently to ensure that the examination was as stress-free as possible. The base of the piglet’s skull was enclosed with the entire palm of the hand, and the piglet’s mouth was opened with the help of pressure from the thumb and index finger at the corner of the mouth. Then, the surface of the piglet’s ground teeth could be palpated with the help of a dental probe. The pulp cavity was considered opened if the probe slipped into the pulp cavity during palpation. When the pulp cavity was intact, a smooth tooth surface was observed. The results were documented on a previously prepared table with an “X” for an opened pulp cavity and with a “0” for an intact pulp cavity.

### Piglet skin lesion scoring and weighing

To evaluate skin lesions in piglets that are typically caused by fighting when establishing a suckling order, a score was developed. The head, ears, trunk, limbs (circumferential increase, skin) and claws of the piglets were each evaluated using a score ranging from 1 to 4 (Table 3). The scores were collected five times during the suckling period, the first time within 24 h after birth before the initial treatment, while the other scorings were performed on each consecutive Monday and Sunday before weaning. For the scoring, the piglets were removed from the farrowing crate and separated in a treatment basket. Then, each piglet was taken individually, and all the aforementioned body parts were examined and scored. For weighing, the individual piglets were placed in a container (bowl, bucket or basket appropriate to the piglet size) standing on the scale (AE Adam MTB 20, Säuglings- und Kleinkindwaage, Felde, Germany). The piglets were individually placed in containers on the scales after lesion scoring. The results were documented in a form worksheet, and the piglet was returned to the sow.

**Table 3:** Piglet skin lesion scores

Score	Lesions
0	no injuries
1	minor injuries (< 5 superficial skin wounds)
2	moderate injuries (> 5 superficial skin wounds or < 5 deep skin wounds)
3	high grade injuries (> 5 deep skin wounds)

### Scoring of the sow’s udder

To evaluate the effects of tooth grinding in piglets on the sow’s udder, the parenchyma, skin and teat of each teat complex were assessed individually, and the results were recorded. The scores ranged from 1 to 4 (Table 4). The sow’s udders were examined before farrowing, in the middle of the suckling period and one day before weaning. The examination was carried out when the sows were standing during feeding. The parenchyma, skin of the udder and teats were separately scored at each sampling. For each time point and each sow, the average score based on all teat complexes was calculated for the evaluation because the distribution was not even enough for a statistical evaluation.

**Table 4:** Sow udder score

Score	Lesions
**Parenchyma**	
1	physiological (no abnormal findings)
2	minor-grade abnormal findings (mildly painful, soft consistency, not increased in warmth, small thickenings/nodules)
3	medium-grade abnormal findings (bulging elastic consistency, coarse-grained, moderately painful)
4	High-grade abnormal findings (coarse consistency, thickenings and nodules present, markedly increased warmth, painful)
**Skin**	
1	physiological (no abnormal findings)
2	minor grade abnormal findings (superficial lesions: Injury to the upper layers of skin, minor redness, possibly minimal bleeding/scabbing)
3	Medium-grade abnormal findings
4	High-grade abnormal findings (deep injury: injury of deep skin layers with redness and scabbing/bleeding, possibly necrotic/purulent)
**Teat**	
1	physiological (no abnormal findings)
2	low-grade abnormal findings (injury to the upper teat skin, minor redness, possibly minimal bleeding/scabbing, functional)
3	medium-grade abnormal findings (injury to the deep teat skin, gaping wound edges)
4	high- grade abnormal findings (teat breakage, no longer functional)
-44	inverted teat

### Piglet and sow behaviour scan-sampling

The behaviour of the piglets before and after tooth grinding was recorded with the help of six cameras (Berghoch, B-A5G-KI, Hartford Electronics GmbH, 44,139 Dortmund, Germany) installed at six randomly selected farrowing pens 2.50 m above the centre aisle and connected to a digital video recorder. A hard drive (Intenso Memory Case, Intenso International GmbH, external hard drive, 6.35 cm-USB 3.0, 49,377 Vechta, Germany) with 1 TB was installed for direct storage of the videos, which was used in exchange with another external hard drive of the same type to store the recordings on an 8 TB external hard drive (Intenso Memory Center, Intenso International GmbH, external hard drive, 8.90 cm- USB 3.0, 49,377 Vechta, Germany). This was connected to a personal computer to view the recordings using a VLC media player (3.0.11). The cameras were aligned with the farrowing pens so that all areas were clearly visible on the recordings. As the availability of cameras was limited, a total of three sows were selected per farrowing group, one from each treatment group. Care was taken to ensure that the sows had varying parity and no previous illnesses. The data of twelve sows were recorded by camera, but only the recordings of 9 sows were available for evaluation since technical problems occurred during the last batch and the recordings were unusable.

The recording started 24 h before the expected date of parturition and ended with the weaning of the piglets. The records of one day comprised the time from 5:00 a.m. until 8:00 p.m. The recordings were saved and evaluated afterwards on a separate computer. Evaluation of the behaviour began on the day of tooth grinding. Two periods of time, before and after tooth grinding, were evaluated on this day for each litter. Each period of time was one hour, and the recording was paused every ten minutes to document the current piglets’ behaviours. This scan sampling resulted in 14 observations per sow for the day of initial care. On all other days of the suckling period, the observation time was set from 8:00 a.m. − 9:00 a.m. and 2:00 p.m. − 3:00 p.m. for all litters according to the same scan sampling method as above. Because the farrowing of the sows did not occur exactly on the same day but the weaning day was the same, the total number of suckling days of the sows varied. The videos were evaluated first for the action of the piglets: (1) Suckling (piglet had a teat of the sow in its mouth and sucked), (2) Sitting (front legs were stretched, and the hind legs lay on the floor); (3) Lying (piglet lay with its chest completely on the floor and rested); (4) Running (piglet stood on all four limbs), (5) Social contact (yes/no, one body part has contact with a body part of another animal sow/piglet). Moreover, the action of the sow was evaluated regarding sitting, lying and standing. The number of piglets showing one of the behaviours at the observation time points was documented on a separate worksheet.

### Statistical analysis

The results of piglet skin lesion scores, piglet weight, sow-udder scoring and behavioural analysis were recorded in a separate data sheet (Microsoft® Office Excel 2016; Microsoft Corporation, Redmond, WA, USA). Statistical analysis for the examination of the teeth for damage due to abrasion, piglet skin lesion scores, weight and sow udder scoring was performed using SAS Enterprise guide, Version 7.1, and SAS software, Version 9.4 (SAS Inst., Cary, NC, USA). The objective of the analysis of the data was to determine whether there was a significant difference in the parameters collected from the sow’s udder and those collected from the piglets between the treatment groups. In all calculations, the significance level was set at *p* < 0.05.

The pulp cave openings were analysed using a logistic mixed model with the fixed effects treatment group, jaw, teeth and the interaction of jaw and teeth and the random effects of sows and piglets nested in sows. *Post hoc* tests were performed with the Tukey-Kramer test.

The distribution of the piglet’s skin scores 1 - 4 was not even, no separate statistical evaluation could be carried out in relation to the degree of lesions. Therefore, the skin score was analysed as a binomial variable (with vs. without skin lesion) using a logistic mixed model with the fixed effects of treatment group, sex, time point, and batch; the interaction between the treatment groups and time point; and the random effects of sow nested in batch and piglets nested in sow. *Post hoc* tests were performed with the Tukey-Kramer test.

The effect of the various factors on piglet’s weight gain was calculated using a linear mixed model with the following fixed effects: treatment group, sex, parity, time point, batch, interaction between treatment group and time point, interaction between parity and time point, PPDS (post partum dysgalactia syndrome) treatment of the sow, and interaction between time point and PPDS treatment of the sow. A random effect was added for sows nested in batches. The repeated examination of the piglets (nested in sows) was incorporated using a heterogeneous autoregressive correlation structure (ARH [1]), i.e., different variances were allowed for different time points. *Post hoc* tests were performed with the Tukey-Kramer test.

For the udder scoring of the sows, descriptive analysis and mixed model analysis were performed using logarithm values of the average scores. A linear mixed model with the fixed effects of treatment group, time point, batch, interaction between treatment group and time point and treatment of the sow was used. Repeated examinations of the sows (nested in batches) were included as repeated measurements with an unstructured correlation matrix for the parenchyma and teat and an autoregressive correlation structure (ARH [1]) for the skin.

Piglet and sow behaviour scan-sampling analysis was performed using the statistical software R 4.1.3 [23]. The behaviour of the piglets was analysed using a logistic mixed model with the fixed effects: treatment group, time point, daytime, behaviour of the sow, the interaction between treatment group and time point, interaction between treatment group and daytime and the random effect of the sow. Four different behaviours of the piglets (suckling, lying, running, sitting) were observed. For the statistical evaluation of piglet behaviour, the probability of occurrence of a particular behaviour was determined. Lying was observed most frequently and was therefore taken as the reference behaviour in the statistical analysis. Four different models were calculated: suckling vs. lying, sitting vs. lying, and running vs. lying.

## Results

### Pulp cave opening in the piglets of different treatment groups

**Fig. 2:**
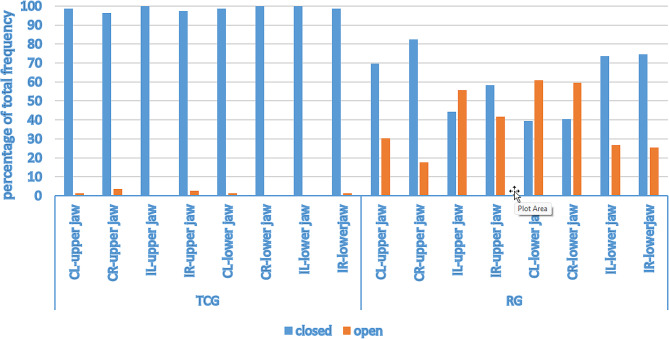
Pulp caves intact or opened after tooth grinding. Tea-cup (TCG) or roller (RG) grinding head. Number of piglets: TCG = 79, RG = 79

In the CG, which did not receive any dental treatment, 79 piglets were examined, and no damage could be observed in the eight front teeth. When examining the front teeth for damage caused by grinding in a subset of 79 piglets in the TCG and 79 piglets in the RG, the proportion of opened pulp caves in the RG differed significantly from that in the TCG (*p* < 0.01). More open pulp caves were found in the RG. In TCG, 10% (*n* = 8) of the piglets had one opened tooth, zero piglets had more than one open pulp, and 90% (*n* = 72) of the piglets had intact teeth. In the RG, 73.42% (*n* = 58) of the piglets had more than one tooth opened, 15.19% (*n* = 12) of the piglets had only one tooth opened, and 11.39% (*n* = 9) of the piglets had intact teeth. A more detailed descriptive analysis of the individual jaws and teeth revealed some differences. In the TCG group, there was no damage to the left Id in the upper jaw or to the right Cd or left Id of the lower jaw. Opened pulp caves were on the right Cd (*n* = 3), left Cd (*n* = 1) and right Id (*n* = 2) in the upper jaw as well as right Id (*n* = 1) and left Cd (*n* = 1) in the lower jaw (Fig. 2). In the RG, opened pulp caves were observed in all teeth. Pulp cave damage mainly occurred in the left Cd and right Cd of the lower jaw, while the pulp cave of the Cd of the right upper jaw was least damaged. Unfortunately, the three-factor interaction (group*jaw*teeth) could not be included in the model due to quasicomplete separation. However, the effect of the two-factor interaction jaw*teeth was statistically significant (*p* < 0.01). More pulp cave openings were observed in the left Cd in the lower jaw than in the left Cd in the upper jaw (significant difference, *p* < 0.01). The left Id in the upper jaw was more frequently damaged than the left Id in the lower jaw (*p* < 0.01, Fig. 2).

### Skin lesion score, body weight and mortality in the piglets of different treatment groups

Skin lesions in a total of 1438 piglets from 110 litters were scored at each of five subsequent time points between birth and weaning.

**Fig. 3:**
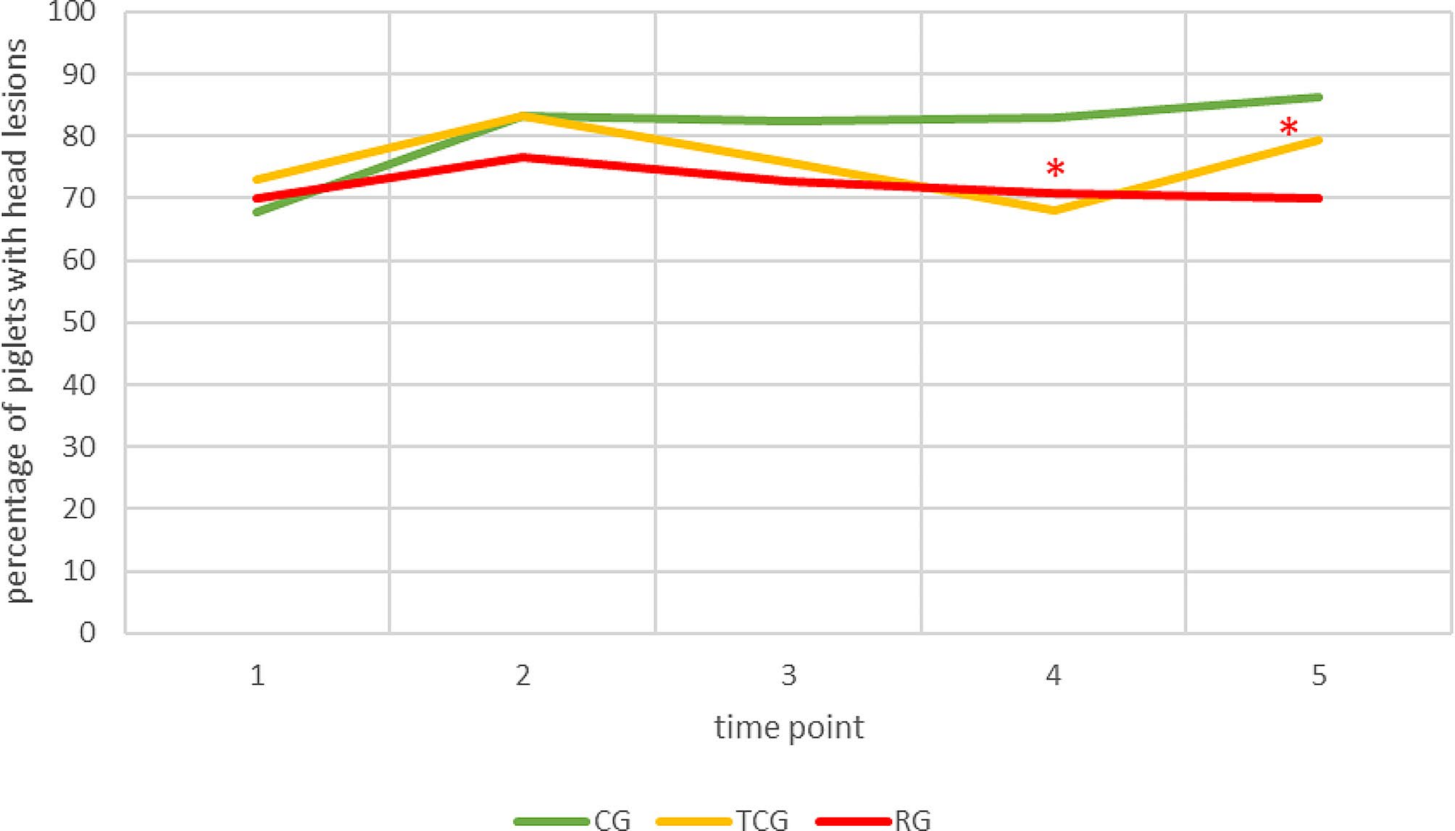
Percentage of piglet skin lesions located on the head. Litters with no tooth grinding (CG) or tooth grinding with a Tea-cup (TCG) or roller (RG) grinding head. Time point 1: within the first 24 h after birth, Time point 2: after one suckling week, Time point 3: after two suckling weeks, Time point 4: after three suckling weeks, Time point 5: day before weaning; Number of piglets: CG = 457, TCG = 523, RG = 458

There was a significant influence of treatment group on the occurrence of lesions on the head (*p* = 0.03). Post hoc tests revealed that the CG and RG differed significantly (*p* = 0.02), with lesions occurring more often in the CG than in the RG. The interaction between treatment group and time point was statistically significant (*p* < 0.01). Before and until (and including) the second time point after tooth grinding (time point 3), there was no significant difference between the treatment groups. At the third time point after grinding (time point 4), the CG and TCG differed significantly (*p* < 0.01). At the fourth time point after grinding (time point 5), the CG and RG differed significantly (*p* < 0.01). In both cases, the proportion of skin lesions in the CG was greater than that in the other treatment groups. Furthermore, no significant effect of treatment was confirmed for lesions on the ears, trunk, limbs or claws of the piglets (Fig.3).

**Fig. 4:**
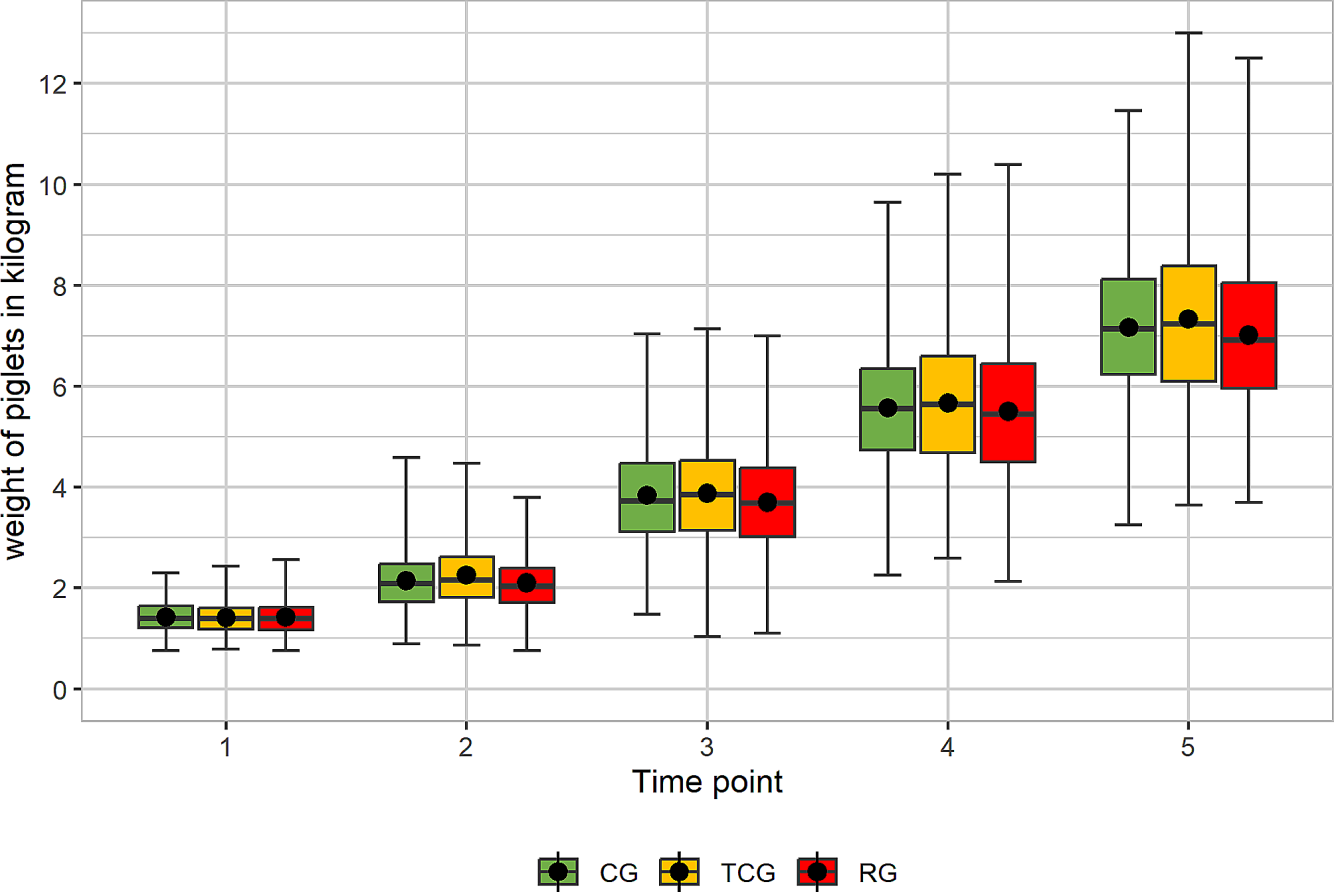
Boxplots for body weight (kg) of piglets belonging to litters with no tooth grinding (CG) or with tooth grinding with the Tea-cup-(TCG) or Roller-(RG) grinding head. Means are indicated by dots. Time point 1: within the first 24 h after birth, Time point 2: after one suckling week, Time point 3: after two suckling weeks, Time point 4: after three suckling weeks, Time point 5: day before weaning. Number of piglets: CG = 552, TCG = 646, RG = 567

To evaluate weight gain, the piglets were weighed at each of the five time points (RG: *n* = 552, TCG: *n* = 646, CG: *n* = 567 piglets). A total of 976 observations, were excluded due to the dropout of piglets that were cross-fostered or died. The first weighing of piglet resulted in starting weights of 1.43 kg (CG) (standard deviation = 0.326), 1.41 kg (TCG) (standard deviation = 0.311) and 1.42 kg (RG) (standard deviation = 0.340). However, no significant difference in piglet weight was found between the CG and TCG or between the CG and RG. At the fifth time point, one day before weaning, the average weights of the piglets were 7.17 kg (CG) (standard deviation = 1.465), 7.33 kg (TCG) (standard deviation = 1.693) and 7.01 kg (RG) (standard deviation = 1.473) (Fig. 4). The weights of the RG piglets were significantly lower than those of the TCG piglets (*p* = 0.02) at the fifth time point. At the first time point, before grinding, there was no significant difference between the treatment groups and the piglets (Fig. 4).

**Fig. 5:**
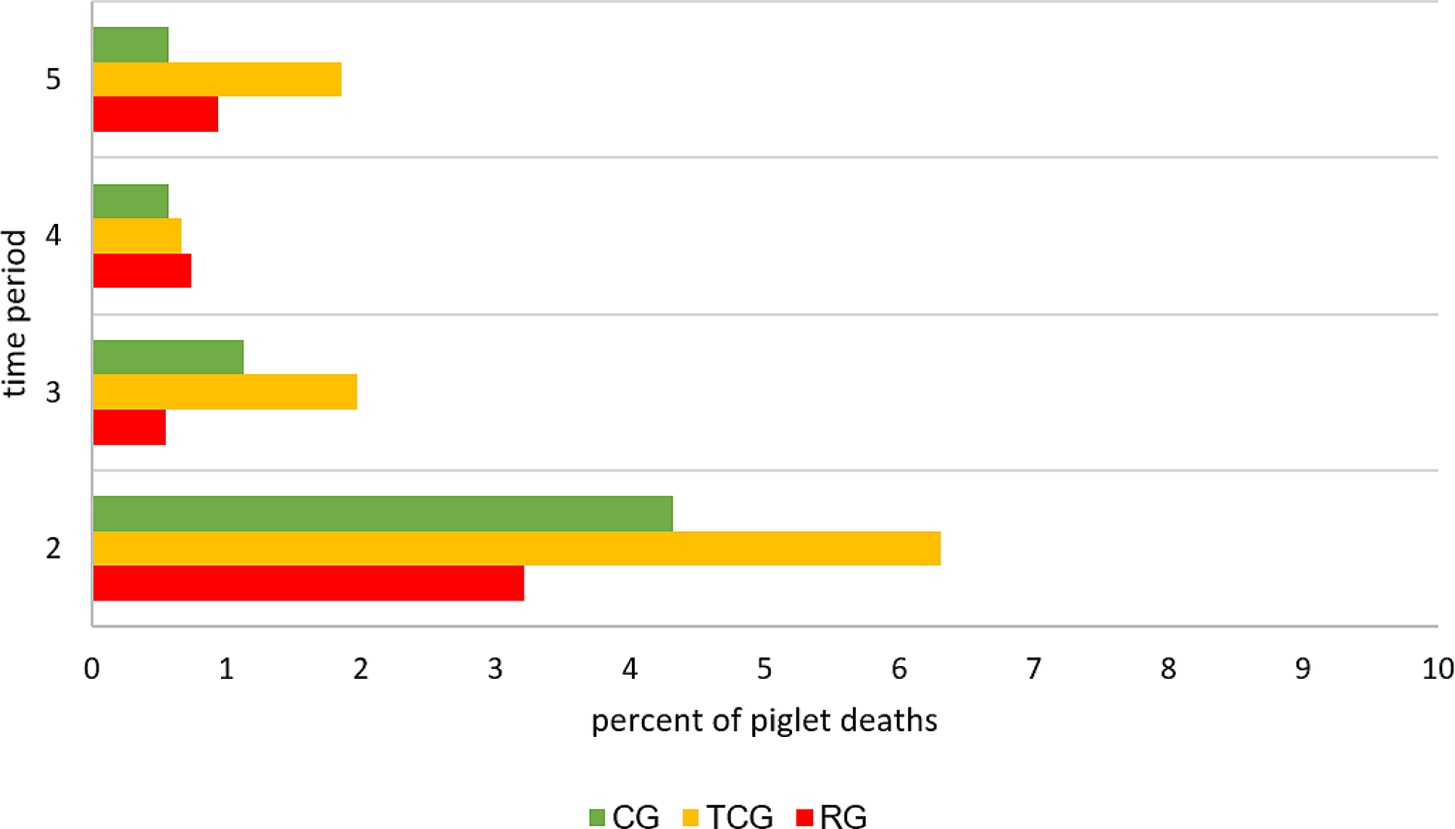
Mortality (%) of piglets. Litter with no tooth grinding (CG) or tooth grinding with a tea-cup (TCG) or roller (RG) grinding head were used. Time period 2: from first score to second score, after one suckling week, Time period 3: from second score to third score, after two suckling weeks, Time period 4: from third score to fourth score, after three suckling weeks, Time period 5: from fourth score to one day before weaning. Number of piglets: CG = 552, TCG *=* 523, RG = 458

The different treatments (TCG, RG and CG) had no significant effects on the mortality of the piglets (CG = 6.48%, TCG = 10.46%, RG = 5.36%). Descriptively, a tendency is evident that there were more losses in TCG over the suckling period. In particular, at the second time point, the losses in the TCG were greater (6.31%) than those in the CG (4.32%) and RG (3.22%) treatments, but no significant differences were detected. A closer look at the distribution of losses in the treatment groups revealed that 41 (60.3%) piglets died during the second observation period in the TCG (CG = 66.6%, RG = 60%, Fig. 5).

### Scoring of the sow’s udder

The average udder scores increased over the suckling period. The average score for the parenchyma and the skin increased by more than 0.2 from the first to the last sampling time and for the teat, an increase of more than 0.1 was observed (Table 5). The effect of time point in all three mixed models was significant (*p* < 0.01) and residuals of parenchyma and teat showed a substantial deviation from normality. However, no significant difference was found between the treatment groups.

**Table 5:** Udder lesions in sow suckling litters subjected to different treatments

	Udder lesions					
	Parenchyma (average score)		Skin (average score)		Teat (average score)	
Treatment*	time 1	time 3	time 1	time 3	time 1	time 3
CG	1.02	1.27	1.39	1.63	1.01	1.18
TCG	1.03	1.40	1.34	1.74	1.02	1.24
RG	1.04	1.20	1.42	1.63	1.01	1.18

* Average score for parenchyma, skin and teat lesions, evaluated before farrowing (Time 1) and at weaning (Time 3), Number of sows: CG (no tooth grinding) = 102, TCG (tooth grinding with a Tea-cup) = 116, RG (tooth grinding with a roller head) = 108

### Scan sampling of piglet behaviour

**Fig. 6:**
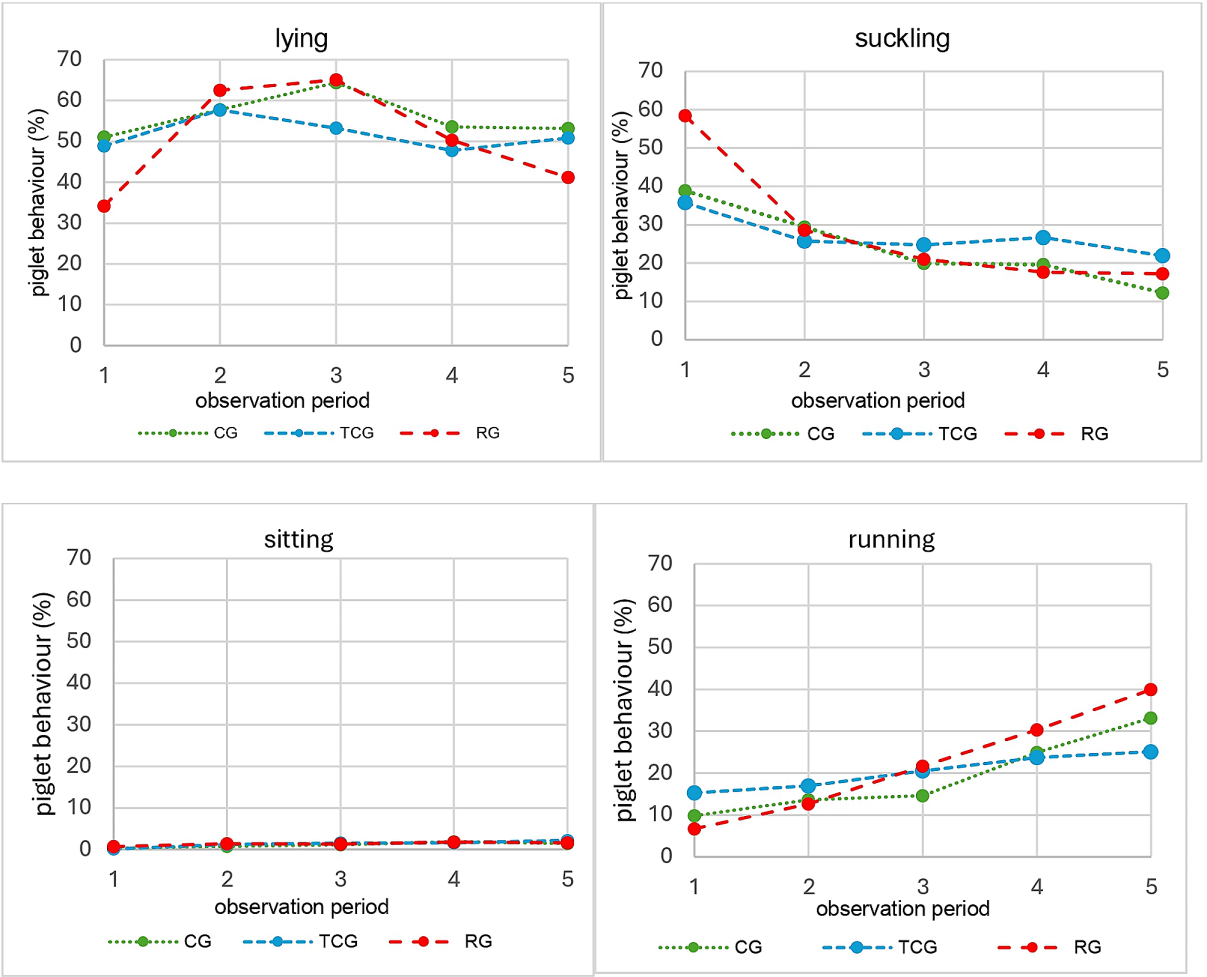
Percentage of piglets within a litter showing different behaviours (lying, suckling, sitting, running) in different treatment groups: no tooth grinding (CG) or tooth grinding with a Tea-cup-(TCG) or Roller-(RG) grinding head. Observations were made from the first day of life, before teeth grinding, until weaning. Number of piglets: CG = 50, TCG = 48, RG = 49

There was no significant effect of the treatment type on suckling behaviour over the entire suckling period (*p* = 0.23). However, there was a significant influence of the interaction between the treatment group and time point of behavioural observation. Pairwise comparisons revealed that in the first observation period, suckling behaviour was significantly different between the RG and the CG and between the RG and the TCG, with piglets in the RG showing more suckling behaviour than those in the other treatment groups. During the fifth observation period, significant differences in suckling behaviour were found for the TCG and CG as well as for the RG and CG, with piglets in the CG showing less suckling behaviour than those in the other two treatment groups (Fig. 6; Table 6).

Sitting behaviour was not significantly affected by the treatment type or by any other variable of the statistical model. For running vs. lying, there was a significant effect of the treatment type over the entire suckling period (*p* = 0.03) as well as of the interaction between treatment type and time point (*p* < 0.01). In the first observation period, significantly greater odds of observing running behaviour instead of lying were found for the TCG than for the CG. The odds of observing running behaviour in the RG during the second observation period were significantly lower than those in the TCG. During the third and fifth observation periods, running piglets were more likely to be observed in the TCG and RG than in the CG. During the third observation period, running piglets were more likely to be observed in the TCG and the RG than in the CG. In the fifth observation period, significantly greater odds of observing running behaviour instead of lying were found for the RG than for the CG and TCG; Fig. 4; Table 6).

No significant differences were found in the model results for piglets’ social contact vs. no social contact.

**Table 6:** Results of pairwise *treatment* group comparisons (suckling/sitting/running vs. lying)

Observation period	TCG* vs. CG				RG vs. CG*				RG* vs. TCG			
	*p*- value	odds ratio	CI lower	CI upper	*p*- value	odds ratio	CI lower	CI upper	*p*- value	odds ratio	CI lower	CI upper
**suckling vs. lying**												
1	0.719	0.771	0.454	1.310	0.002	2.118	1.262	3.555	< 0.001	2.748	1.611	4.688
2	1.000	0.840	0.536	1.316	0.481	0.768	0.490	1.204	1.000	0.915	0.583	1.436
3	0.085	1.510	0.963	2.368	0.729	1.245	0.794	1.954	0.918	1.245	0.526	1.294
4	0.054	1.560	0.994	2.449	1.000	1.036	0.658	1.631	0.091	0.664	0.422	1.044
5	0.003	1.923	1.193	3.098	< 0.001	2.144	1.325	3.471	1.000	2.144	0.700	1.776
**sitting vs. lying**												
1	1.000	0.894	0.045	17.785	0.295	4.263	0.523	34.781	0.496	4.769	0.322	70.646
2	0.436	1.630	0.730	3.641	0.341	1.691	0.763	3.747	1.000	1.037	0.488	2.205
3	0.511	1.530	0.729	3.211	1.000	1.164	0.551	2.461	1.000	0.761	0.366	1.582
4	1.000	0.942	0.464	1.914	1.000	0.862	0.428	1.735	1.000	0.914	0.447	1.869
5	0.411	1.635	0.741	3.609	0.916	1.415	0.629	3.182	1.000	0.865	0.410	1.827
**running vs. lying**												
1	0.003	1.938	1.204	3.119	1.000	1.162	0.671	2.013	0.065	0.600	0.352	1.022
2	0.195	1.214	0.944	1.560	0.206	0.822	0.635	1.064	< 0.001	0.677	0.525	0.874
3	< 0.001	1.556	1.220	1.984	< 0.001	1.553	1.220	1.976	1.000	0.998	0.787	1.266
4	1.000	1.077	0.851	1.362	0.352	1.164	0.923	1.468	1.000	1.081	0.855	1.366
5	0.109	0.801	0.621	1.032	< 0.001	1.536	1.196	1.973	< 0.001	1.919	1.499	2.457

* Number of piglets: CG (no tooth grinding) = 50, TCG (tooth grinding with a Tea-cup grinding head) = 48, RG (tooth grinding with a roller grinding head) = 49, Observation from first day of life, before tooth grinding, until weaning; (*p* value adjustment method: Bonferroni)

## Discussion

In most conventional German piglet production farms, resection of the tip of teeth that are fully present at birth (e.g., Id3 and Cd) is routinely performed to avoid injuries inflicted by piglets among themselves and to the sow’s udder. The purpose of this study was to analyse various effects on piglets after no tooth grinding (CG), tooth grinding with a recently developed Tea-cup grinding head (TCG) and the conventional method of grinding with a diamond roller grinding head (RG). The study must be critically considered, as it only concerns a single herd.

During the dental resection of piglets, damage occurs to the teeth and surrounding tissue [9, 14]. In a study by Hessling-Zeinen (2014), the pulp cavity was opened on at least one tooth in 90% of the piglets when the teeth were ground with a roller grinding head. In total, 45% of the teeth had an opening of the pulp cavity [9]. The Tea-cup grinding head was developed to minimise tooth lesions by grinding every tooth individually. In the present study, examination of the teeth for pulp opening confirmed that grinding with the roller head resulted in significantly more pulp opening than grinding with the Tea-cup grinding head. This confirms the results of the study of Ellert (2017) showing that the Tea-cup grinding head can be used to minimise the number of pulp openings caused by tooth resection [22]. In addition, a significant difference was found between the opening of the teeth in the upper and lower jaw. This could be related to the person performing the tooth resection. Since the tooth resection was always performed by the same person, it is not possible to determine whether different handling has an influence on the lesions on the tooth. In order to assess this, further tests would have to be carried out in which the person carrying out the test varies.

The aim of tooth resection is to minimise skin lesions on the piglets and on the sow’s udder and thereby to improve animal welfare and ensure high weight gain in the piglets as well as sufficient milk production by the sow. Previous studies have reported a reduction in the number and severity of facial lesions in piglets when teeth are clipped [5–7, 14–16, 18] or ground [7, 14]. However, no effect was found in another study [24]. In the present study, significantly more piglets in the CG than in the RG had head lesions. This finding generally confirms the hypothesis that dental resection can result in fewer skin lesions on the piglets’ heads. However, the TCG and CG did not differ significantly in the number of head lesions, except at the third time point. Possibly this effect is due to residuals of sharp edges not removed when the resection is really limited to the tip of each tooth or avoiding the pulp opening in most piglets is resulting in less pain associated with more fighting for access to teats. Basically, the lesions on the head of the piglets must be contrasted with the lesions on the teeth. It is debatable whether the visible superficial lesions on the head of the piglets are more painful than the invisible deep, persistent lesions on the tooth. To answer this question, it is essential to carry out further investigations into the pain behaviour of the animals [8].

To assess the consequences of the lesions on the welfare of the piglets, the weight trajectory was documented. Piglet weight gain is an important metric for assessing the productivity of the piglets and, to a certain degree, may indicate their well-being. The weight trajectory of the piglets over the observation period showed a significant difference between the treatments. Before treatment, there was no significant difference in the body weights of the piglets. At weaning, the piglets in the TCG group weighed significantly more than the piglets in the RG group. There was an average difference of 158 g body weight between the TCG and CG groups, but this difference was not significant. This treatment effect contradicts much of the literature, which reports no effect of tooth clipping [6, 16, 18, 19] or tooth grinding [6, 19] on the weight gain of piglets. Some studies have reported lower weight gains within the first [5], second and third [1, 20] weeks after tooth grinding. However, it needs to be considered that the Tea-cup grinding head was used for the second time in this study. The tendency towards a positive effect on body weight in the TCG might have been influenced by enhanced milk intake, which is directly related to piglet welfare and weight gain. With regard to suckling behaviour, a difference between the TCG and CG groups could only be determined at the end of the suckling period in this study. These results indicate that the feed intake of the piglets in the TCG was better than that of the piglets in the other treatment groups (RG, CG). The study could not conclusively clarify why the weight gain of the TCG was higher compared to the other groups, especially at weaning. Since tooth lesions are one of the decisive differences between the RG and TCG in the study, it can be assumed that the opened teeth have an influence. It is possible that the permanent damage to the teeth in the RG reduced feed intake long after treatment, whereas the piglets in the TCG showed only a small number of changes. In the CG group, there are no injuries to the tooth, but the increased number of facial injuries can also lead to reduced feed intake here.

In this context, these study findings contradict those of previous investigations, as the CG had a greater body weight at weaning than did the RG. Body weight at weaning is an important factor for farmers in terms of weight gain after the suckling period [25].

In terms of mortality, the study showed that more suckling piglets died in the TCG than in the other treatment groups, but this difference could not be statistically confirmed. Most studies have found no significant effect on mortality in relation to tooth clipping [5, 6, 16, 18] or grinding [6]. Accordingly, no relationship between mortality and treatment can be established. In the study, no evidence could be found that animals died due to tooth openings.

The lesions observed on the udder of the sow (parenchyma, skin, teat) did not significantly differ among the three treatments. This confirms the findings of previous studies, performed in outdoor systems, of lesions in the udder of sows in herds where routine tooth clipping was carried out, and the results were compared to those of treatment groups without tooth clipping [16, 17]. However, the results of this recent study contradict those of other studies in which fewer injuries were found when teeth were clipped [14] or ground [7].

The behaviour of litters was analysed as a further indicator of animal welfare. Interpreting the results, it must be considered that the small sample size of nine sows can only show a tendency. Furthermore, the behaviour of the litters differed significantly even before tooth resection, and consequently, there were no good baseline data. The differences could be related to the fact that the number of observations in period one, before treatment, was substantially lower than the number of observations after treatment in period two. When comparing the behaviour before and after treatment, no significant difference was found. This suggests that tooth resection has no obvious visible short-term effect on the immediate behaviour of piglets. Other studies have shown that, especially in the first few days after tooth clipping, playing/fighting behaviour decreased significantly compared to that in the control group [26],but this was not confirmed in the present study. It is debatable whether the handling of the piglets, which was carried out equally in all treatment groups, was more of a stress factor than tooth resection itself. This isolation stress, which occurs when the piglets are separated from the sow, has already been recorded in previous research [13, 27]. Compared with animals of the control group, all animals of the experimental groups showed significant changes in behavioural response, cortisol and ACTH release and corticosteroid receptor expression in the hypothalamus [27]. In the present study, the duration of isolation differed among the treatments. As tooth grinding with the Tea-cup grinder takes twice as long as with the roller grinding head [22], the isolation and handling of each piglet was longer in the TCG while the CG piglets received no handling or treatment in the respective time. It should be assumed that the TCG animals that have been isolated for a longer period of time will show a change in behaviour. Nonetheless, no significant differences in the behaviour of the piglets were detected the first time after grinding. Based on these results, it can be assumed that the duration of isolation and the treatment have no major effect on the piglets or their pain perception. Due to the small sample size in this study, this should be confirmed in further studies.

Some significant differences were found between the behaviours and treatments. With regard to the results already mentioned, it is clear that the TCG exhibited the suckling behaviour most frequently over the suckling period. This is consistent with the significant difference in weaning weight. None of the other significant differences could be correlated with the previous results.

However, there were significant differences in behaviour between the treatment groups at the end of the suckling period. This suggests that the type of treatment might have a long-term effect on the behaviour of the piglets; however, this effect needs to be further investigated. Another study has already shown that grinding and clipping cause long-lasting pain. However, it was also found that further investigations are required to determine the extent of long-term damage [8]. 

Performing tooth resection involves additional work. This effort must have added value for the farmer in terms of productivity. The time taken to grind teeth with the roller grinding head was determined to be 9.6 s in a recent study [22]. In comparison, resection with the Tea-cup head took twice as long [22]. The time required was not examined in this study, which is why no comparative values are available. In view of the results already obtained, in the CG, these expenses can be disregarded, and the time required between the RG and TCG can be classified as twice as high. Therefore, the additional benefit in the TCG group should be significantly greater than that in the other treatment groups. For a better evaluation of the possible benefits of tooth grinding by the Tea-cup head, the long-term effects, including the nursery and fattening periods, need to be carefully evaluated in further studies. Moreover, the effect should be evaluated on the basis of a larger number of herds.

In addition to the choice of method, it should be carefully checked whether there is a need tooth resection at all. Tooth grinding should be limited to individual litters but it remains difficult for the farmer to recognise the point at which tooth grinding becomes necessary for the individual litter. For this purpose, it would be useful to develop criteria on the basis of when an individual decision can be made as to whether teeth should be resected. Moreover, farmers should be informed about the damage that inevitably occurs when using a roller head grinder. Without this information, farmers would only consider clearly visible skin lesions in their decision while not taking into account any hidden or hardly recognisable pulp openings.

## Conclusion

In this study, the effects of different tooth grinding procedures on piglets and sows were investigated. Tooth lesions (pulp openings) due to resection with the roller grinding head were found in most of the piglets (88.6%) and still in 10% of the piglets treated with the Tea-cup grinding head. It can be concluded that tooth resection should always be critically scrutinised, as it is often resulting in pulp openings and does not solve the basic problem that often lies in management. Other approaches, such as improving sow milk production, reducing litter size, and litter management, should be considered before tooth resection is carried out. When performing tooth resection is indispensable, the use of the Tea-cup head is recommended to avoid the large number of pulp opening inevitably induced by the roller grinding head. Farmers should also be better informed about tooth damage caused by tooth resection to make them aware that these invisible/barely visible lesions are likely an entryway for infectious agents. Moreover, there is still a large gap in studies on the long-term consequences of dental injuries and their effects on animal performance. In addition, the possibility of measuring pain in during the tooth resection and as a consequence of pulp opening is still limited, which is why this point has not been sufficiently clarified in many respects.

## Data Availability

The datasets generated and analysed during the current study are not publicly available because the farmer has given written consent stipulating that the data may not be passed on to third parties but are available from the corresponding author upon reasonable request.
